# Usability Evaluation Methods Used in Electronic Discharge Summaries: Literature Review

**DOI:** 10.2196/55247

**Published:** 2024-09-12

**Authors:** Wubshet Tesfaye, Margaret Jordan, Timothy F Chen, Ronald Lynel Castelino, Kamal Sud, Racha Dabliz, Parisa Aslani

**Affiliations:** 1 The University of Sydney School of Pharmacy Sydney Australia; 2 The University of Queensland School of Pharmacy Brisbane Australia; 3 The University of Sydney School of Medicine Sydney Australia; 4 Nepean Kidney Research Centre, Nepean Hospital Sydney Australia; 5 Australian Commission on Safety and Quality in Health Care Sydney Australia

**Keywords:** electronic discharge summaries, usability testing, heuristic evaluation, heuristics, think-aloud, adoption, digital health, usability, electronic, discharge summary, end users, evaluation, user-centered

## Abstract

**Background:**

With the widespread adoption of digital health records, including electronic discharge summaries (eDS), it is important to assess their usability in order to understand whether they meet the needs of the end users. While there are established approaches for evaluating the usability of electronic health records, there is a lack of knowledge regarding suitable evaluation methods specifically for eDS.

**Objective:**

This literature review aims to identify the usability evaluation approaches used in eDS.

**Methods:**

We conducted a comprehensive search of PubMed, CINAHL, Web of Science, ACM Digital Library, MEDLINE, and ProQuest databases from their inception until July 2023. The study information was extracted and reported in accordance with the PRISMA (Preferred Reporting Items for Systematic Reviews and Meta-Analyses). We included studies that assessed the usability of eDS, and the systems used to display eDS.

**Results:**

A total of 12 records, including 11 studies and 1 thesis, met the inclusion criteria. The included studies used qualitative, quantitative, or mixed methods approaches and reported the use of various usability evaluation methods. Heuristic evaluation was the most used method to assess the usability of eDS systems (n=7), followed by the think-aloud approach (n=5) and laboratory testing (n=3). These methods were used either individually or in combination with usability questionnaires (n=3) and qualitative semistructured interviews (n=4) for evaluating eDS usability issues. The evaluation processes incorporated usability metrics such as user performance, satisfaction, efficiency, and impact rating.

**Conclusions:**

There are a limited number of studies focusing on usability evaluations of eDS. The identified studies used expert-based and user-centered approaches, which can be used either individually or in combination to identify usability issues. However, further research is needed to determine the most appropriate evaluation method which can assess the fitness for purpose of discharge summaries.

## Introduction

The adoption of digital health platforms for collecting, sharing, and analyzing health information has shown positive associations with improvements in health care quality, service delivery, and clinical benefits including patient safety [1-4]. Electronic health records (eHRs) have become essential in acute care facilities as they enable the collection, sharing, and analysis of patient-related information, facilitating communication within and across health care settings. However, despite the substantial growth in the digitalization of health information exchange platforms, the complexity of many systems used by health care providers often poses challenges in achieving interoperability across different settings [5,6].

Differences in electronic systems used across different health settings can affect the exchange of relevant patient health and clinical information, especially during transitions of care or clinical handover [7,8]. Evidence indicates that the suboptimal communication between hospitals and external health care providers leads to discrepancies in medication records, duplication of tests, and avoidable delays in service provision, especially affecting vulnerable populations including those with low levels of health care literacy [9,10]. Hence, a coordinated health system with improved health information exchange, usability, and interoperability across health facilities and settings has significant potential to improve postacute care transition and overall patient safety [11].

Hospital discharge is a high-risk event where inaccurate or delayed transfer of clinical information, including medication plans, can significantly risk patient safety and cause medication-related issues [12,13]. Therefore, the clinical handover at the point of hospital discharge is a crucial step in patient care that determines the quality of care and patient safety. The introduction of electronic discharge summaries (eDS) has greatly improved the timely transmission of information to relevant stakeholders, mainly those in the primary care setting [8,14]. eDS, defined as “an end-to-end electronic transfer from the hospital to the community, using a secure messaging system, with the information populated using both pre-populated fields and manual transcription” [15] has seen increased adoption over the past decades. However, to further improve the quality of care and reduce communication delays between health settings, it is crucial that eDS should be user friendly [16]. This will help in minimizing avoidable patient harm incidents caused due to usability issues.

Usability is generally defined as “the effectiveness, efficiency and satisfaction with which specified users achieve specified goals in particular environments” [17]. In the context of electronic systems, usability refers to whether the system is useful, usable, and satisfying for the intended users to enable completions of intended tasks in certain sequences [18]. Evidence indicates that there are several usability issues identified with eHRs, such as those related to data entry and alerts, interoperability issues, display, automation, and workflow [16]. These usability problems in addition to affecting the implementation of such systems have implications for patient safety such as medication error and use of inappropriate medication doses [16,19]. Evaluation of systems used to prepare eDS provides an opportunity to identify and improve usability issues with existing systems. Usability evaluation involves assessing performance, efficiency, and satisfaction of electronic interfaces and can identify usability issues with eHRs to thereby propose interventions to improve designs of interfaces, their learnability, and service efficiency [20]. While various international organizations have developed and provided guidelines on the content, form, and presentation of eDS [21-27], less is known about the usability of eDS and systems used to display eDS and their potential impact on quality of care.

Evidence from systematic reviews have identified a range of usability evaluation techniques applied broadly to eHRs, which include heuristic evaluation, cognitive walkthrough, think-aloud, user-testing, observation, coupled with use of questionnaires and interviews to assess participants’ perspectives and satisfaction [20,28]. However, there is limited evidence on the usability evaluations applied specifically to eDS. Therefore, the aim of this literature review was to identify the usability evaluation techniques that have been used to assess the usability of eDS.

## Methods

This literature review is reported in accordance with the PRISMA (Preferred Reporting Items for Systematic Reviews and Meta-Analyses) guidelines [29].

### Literature Search

We searched PubMed, CINAHL, Web of Science, ACM Digital Library, MEDLINE, and ProQuest databases from their inception until Jul 2023. The main concepts used for developing our search strategy included the following and are tailored for the individual databases. *Concept 1*: “usability evaluation” OR “usability testing” OR “usability test” OR “usability engineering” OR “usability inspection” AND *Concept 2*: (“discharge summar*” OR “discharge communication” OR “continuity of care” OR “transfer of care” OR “clinical handover” OR “electronic discharge” OR “patient discharge”).

To capture unpublished and unindexed documents, a gray literature search was conducted using Google Scholar and via a range of governmental and health authorities’ websites and guidelines. Reference lists of included studies were also manually searched to identify further eligible studies or government reports which may have been missed during our search. The full search strategy for all databases including gray literature sources is presented in Multimedia Appendix 1.

### Study Selection

Search results were screened for eligibility following predefined inclusion and exclusion criteria. The retrieved studies were exported to EndNote and subsequently transferred to Covidence [30]. After removal of duplicates, the remaining documents were screened using title, abstract, and full-text by 2 independent reviewers (WT and MJ), with disagreements resolved via discussion until consensus was reached.

### Eligibility Criteria

We included studies that used usability evaluation of eDS or discharge communication or those that evaluated usability issues of eHRs used to prepare an eDS and may also have implication or relevance for eDS. The relevance of eHRs for inclusion was determined based on whether the included studies assessed electronic system interactions without explicitly mentioning eDS (eg, cross-facility health information exchange) or were using an electronic platform that is also known to have an eDS component (eg, My Health Record—an Australian digital platform containing a secure web-based summary of key patient health information, where health care providers can access the system to view and upload information). We also considered studies that focused on electronic health information to patients, with the aim of assessing usability of such information to improve care after discharge. Quantitative, qualitative, and mixed methods studies were all eligible for inclusion.

Studies that evaluated the effectiveness of transfer of care tools or interventions on quality of care or patient outcomes but did not include usability evaluation of eDS were excluded. Studies addressing the use of tools without any usability assessment were also not the focus of this review. Publications in languages other than English were excluded. Finally, we also excluded protocol studies without any preliminary findings.

### Operational Definitions

#### Discharge Summary

A range of information about events during care by a provider or organization, with the goal to provide relevant patient, clinical, and administrative information that enables a continuity of care upon patient’s discharge from hospital [21]. While our primary focus is on discharge summaries, we have expanded our scope to include studies addressing usability issues with electronic discharge instructions or information provided to patients or other health care professionals. This was mainly done to understand and address the information needs and preferences of patients during their transition across different types of care.

#### Electronic Discharge Summary

Refers to a computerized form of discharge summary or instructions typically generated within electronic health records used in tertiary care.

#### Usability (of eHRs)

Refers to whether the electronic system is useful, usable, and satisfying for the intended users to enable completions of intended tasks in certain sequences [18].

#### End User

The user of the electronic interfaces, who could be health professionals (eg, physicians, nurses, pharmacists, etc) or consumers (patients or their caregivers).

### Data Extraction and Synthesis

We extracted the following information from included studies: study characteristics (authors, publication year, and country), characteristics of end users or participants targeted, study design used (eg, mixed methods, qualitative, etc), usability evaluation method used (eg, questionnaires, interviews, heuristic evaluation, etc), study outcomes reported, and conclusions and limitations. These data were extracted from included studies using a standardized data extraction format that was modified from the Joanna Briggs Institute’s manual for evidence synthesis [31], which can be found in Multimedia Appendix 2. Due to the nature of the included studies or heterogeneity of study participants and findings, quantitative analysis or meta-synthesis was not possible; however, we conducted a systematic narrative synthesis of the major study findings and their implications.

## Results

### Characteristics of Evidence Source

**Figure 1:**
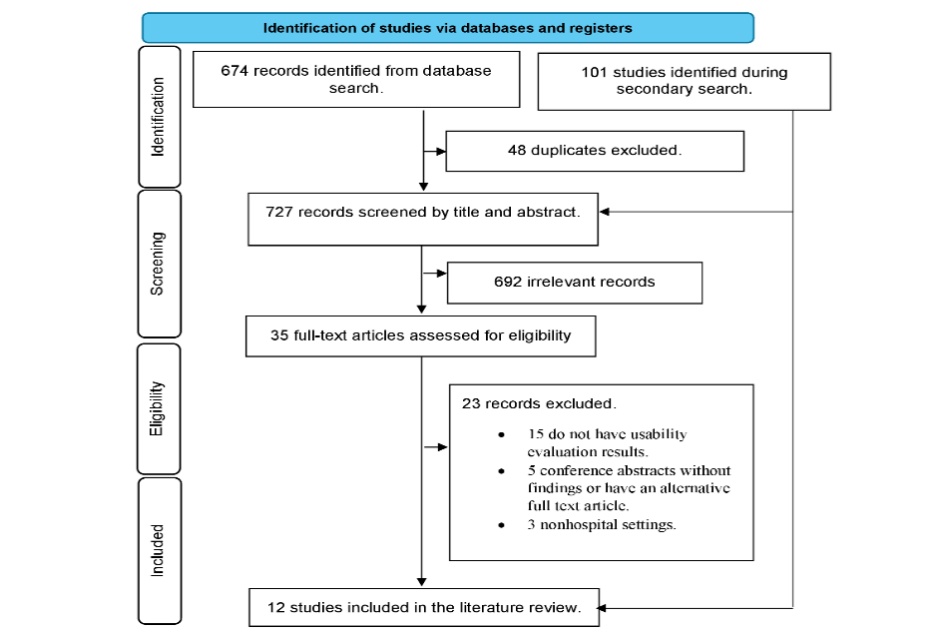
Flow diagram for study selection process.

Our search identified a total of 775 records (see PRISMA flowchart in Figure 1). Of these, 34 were relevant for full text review. After removing duplicate and irrelevant records, 12 studies met the eligibility criteria and were included in this review [32-43].

The studies were conducted in the United States (n=5) [32,36,38-40], Australia (n=3) [34,41,42], Germany (n=2) [33,35], and one each from Canada [37] and France [43] and had used qualitative (including document review and semistructured interviews) or mixed methods (n=9) [32,33,35,37-39,41-43], or observational (n=2) [34,36] methods. One document was a thesis containing a study that used experimental and survey methods and presented some findings on usability testing [40]. Table 1 presents the key characteristics and major findings of included studies, while the detailed study findings are summarized in Multimedia Appendix 2.

**Table 1:** Key study characteristics and major findings.

Study	Country	Study design	Participants	Primary aim	Target of the usability evaluation	Major findings
Barton et al [32]	United States	Qualitative evaluation	Emergency medicine physicians, nurses, geriatrician	To assess a method for integrating diverse expertise such as clinical, patient, care partner, and IT, in the evaluation of patient-facing emergency department after visit summary.	eDS^a^	Identified usability issues related to readability, comprehensibility, and content organization, highlighting the need to integrate experts’ perspectives during design.
Busse et al [33]	Germany	Mixed methods (Qualitative evaluation and observational)	Pediatric palliative care health care professionals	To evaluate how potential users from the pediatric palliative care setting perceived an electronic cross-facility system.	Both contents of cross-facility medical records and the system used for presentation	Identified critical need for data transfer automation and suggested improvements in search functions and visualizations.
Doyle et al [34]	Australia	Exploratory mixed methods	Parents of children and physicians	To understand parent and clinician experience of discharge communication and engagement in clinical research.	System used for presenting electronic discharge instructions	High success rates and satisfaction scores were observed for both mobile and desktop interfaces, with most tasks completed successfully.
Kernbeck et al [35]	Germany	Qualitative observational	Pediatric palliative care professionals	To evaluate the acceptance of the medication module from potential users’ perspective and to involve them in the development process.	Both contents of cross-facility medical records and the system used for presentation	Identified usability issues related to performance expectancy and learnability, emphasized clarity, and reduced cognitive load.
Naik et al [36]	United States	Observational (user-centered)	People with colorectal cancer	To transform physician-centered discharge warnings into patient-friendly format using health literacy and usability heuristics standards and cognitive interviews.	Both eDS contents and the system used for presentation	Identified inconsistencies in content presentation and readability, highlighted importance of a patient-centered design.
Soto et al [37]	Canada	Mixed methods study	General practitioners, family medicine residents	To improve health information exchange and use of clinical information for decision making.	System used for eDS presentation	Identified usability issues related to drug prescription and medication list visualization.
Tremoulet et al [38]	United States	Qualitative evaluation	Human factors experts, medical professionals	To conduct heuristic evaluation to identify potential usability problems and their level of severity.	Both eDS contents and the system used for presentation	Identified usability issues related to content, comprehensibility, readability, presentation, and organizational aspects of medical documents.
Tremoulet et al [39]	United States	Literature review with mixed methods study	Primary care physicians, nurses, nursing and medical directors, social workers, transition-of-care nurses	To provide insight into how existing acute care eDS support outpatient providers in the coordination of care of older adults.	eDS	Identified usability issues affecting care coordination, emphasized need for standardization of discharge summaries.
Vaigneur [40]	United States	Experimental and survey	Novice readers (caregivers) of discharge instructions	To examine the impact of adjusting readability level of discharge instructions on user comprehension and recall.	eDS	High readability discharge instructions received more attention, better comprehension, and reduced mental demand compared to low readability instructions.
Walsh et al [41]	Australia	Qualitative evaluation	My Health Record users	To identify potential usability issues within My Health Record focusing on eHealth literacy.	Both contents of health information summary and the system used for presentation	Identified usability violations and problems related to language use, website navigation, design elements, and registration processes.
Walsh et al [42]	Australia	Qualitative evaluation	My Health Record users	To identify usability issues with My Health Record through an updated heuristic evaluation.	Both contents of health information summary and the system used for presentation	Identified violations of usability heuristics and highlighted unmet needs for individuals with low eHealth literacy.
Watbled et al [43]	France	Mixed methods	Human factors experts, medical professionals	To apply a combination of methods for longitudinal usability evaluation throughout the system development lifecycle and to identify causes of usability flaws.	System used for presentation of eDS	Identified multiple usability flaws in voice recording systems and emphasized thorough analysis and context-specific evaluations.

^a^eDS: electronic discharge summaries.

### Usability Evaluation Methods and Targets

Over half of the included studies [32,36-39,41-43] used a heuristic evaluation method alone or in combination with other methods (Table 2). The method by Nielsen et al or its modified versions [44,45] was the most used heuristic evaluation approach among the included studies [32,36,38,39,41,42]. Watbled et al [43] reported a modified version of the heuristic usability evaluation method known as heuristic walkthroughs.

**Table 2:** Usability evaluation techniques used.

Author	Heuristic evaluation	Think-aloud	Laboratory testing (*in situ* observation, eye-tracking)	Questionnaire (system usability survey)	Interview	Remote evaluation
Barton et al [32]	✓					
Busse et al [33]		✓			✓	✓
Doyle et al [34]		✓	✓	✓		
Kernbeck et al [35]		✓			✓	
Naik et al [36]	✓		✓			
Soto et al [37]	✓	✓			✓	
Tremoulet et al [38]	✓					
Tremoulet et al [39]	✓			✓	✓	
Vaigneur [40]			✓	✓		
Walsh et al [41]	✓					
Walsh et al [42]	✓					
Watbled et al [43]	✓	✓				

User testing methods such as think-aloud [33,35], eye-tracking and *in situ* observation techniques [40,43] were also used for usability evaluation of eDS systems. A combination of evaluation methods (eg, heuristics with think-aloud technique or use of think-aloud method along with a questionnaire) were also used in certain instances [40,43]. Questionnaires like the system usability survey (SUS) [38-40] and semistructured interviews [33,35,37,39] were also used by multiple studies together with other usability evaluation approaches to assess satisfaction and perception of system users. Remote evaluation (via a Zoom-based videoconference) was successfully applied in 1 study [33].

The usability evaluation studies focused on different participant categories. In the heuristic evaluation, the studies mainly involved experts who assessed usability of interface design, while others focused on either end users or a combination of experts and end users. The targeted end users included clinicians, medical secretaries, nurses, patients, or caregivers, while the experts were human factors experts [36,38,43] and domain experts (people with knowledge of broader health system and are experienced users of My Health Record) [41,42].

### Summary of Major Findings

The included studies identified several usability problems with varying degree of severity in both the eDS as well as the systems used to prepare and display eDS. While some studies focused on the usability of eDS, such as content, comprehensibility, structure, and readability issues [32,36,38-40], other studies evaluated the usability of eDS systems from presentation, design, and ease of use points of view [33,35,41-43]. The studies used different usability metrics such as usefulness, system efficiency, learnability, performance, and satisfaction when evaluating the usability of the targeted systems [33-35,37,38,40]. While the use of heuristic evaluation identified several organizational, layout, and formatting-related usability issues with different systems used to host eDS, the combined approach of using heuristic walkthroughs with user testing proposed by Watbled et al [43] tended to identity more severe problems and also highlighted their potential negative impact. These included issues related to error management, workload, and compatibility. These issues could lead to serious outcomes, such as prolonged deadlines for task completion, mistakes in patient identification, and inadequate error detection by users [43].

Studies that used heuristic evaluation overall identified several content, comprehensibility, readability, and structural usability flaws [32,33,36,38,40]. Visualization and presentation problems (eg, visualization of medication list or diagnosis and clarity and readability of medication documentation) were among the domains identified to have the highest number of usability problems and may have an impact on patient comprehension and safety [32,33,35,37,38]. Further, design readability and layout issues were identified to have an association with longer duration of screen gazes, affecting comprehension of discharge instructions [40]. One study reported that less display fragmentation and data entry requirements can reduce the cognitive load of user, confusion, and usability concerns [35]. Similarly, a study that used the eye-tracking method demonstrated that improving readability and layout was associated with less mental demand [40].

Concerns with language use, interface layout, and lack of audio-visuals were identified as common usability flaws in Australian studies that used usability issues with My Health Record, with implications for people with low electronic health literacy [41,42]. Another Australian study that assessed user satisfaction using the SUS questionnaire highlighted high acceptability of a digital discharge communication tool, with consumers and clinicians reporting high satisfaction scores on the mobile (94%) and desktop (93%) interfaces, respectively [34].

One study, which involved an information technology expert, assessed the likelihood of addressing usability issues for a patient-facing emergency department visit summaries [32]. The study reported that nearly half of the usability issues identified were difficult to address (31/76 issues). These are issues with some information originating from different service vendors or when an eHR vendor was responsible for providing parts of the discharge summaries (eg, headers, content, and order of sections).

## Discussion

This review summarizes study findings on usability evaluation approaches used to assess eDS and eDS systems. The limited published evidence revealed the use of heterogenous usability evaluation techniques spanning from one conducted by experts to laboratory and user testing to the use of questionnaires and interviews. Broadly, our findings highlight that the use of heuristics (expert based) and think-aloud (user centered) were the most used methods for evaluation of eDS and eDS systems. Other techniques like eye-tracking, direct observation, questionnaire- and interview-based evaluations were also used in combination with either heuristic or think-aloud approaches.

Heuristic evaluation method, consistent with previous findings on eHRs [28], was used by most of the included studies for evaluation of eDS and eDS systems usability. This technique typically involves the application of a procedure including 3-5 experts to independently apply a set of best practices design (referred as heuristics) to identify usability flaws with system interfaces [44]. The heuristics used in evaluation are either defined *a priori* by experts or are derived from standard guidelines like the ergonomic criteria [46], which has 8 main domains around guidance, workload, explicit control, adaptability, error management, consistency, significance of codes, and compatibility.

Our review indicates that heuristic evaluation method can successfully identify a range of usability issues around readability, comprehensibility, organization, and content of eDS interfaces [38,39]. In addition to identifying usability flaws with user interfaces, heuristic evaluation also enabled assessment of the severity of usability problems. The severity of usability problems is often rated based on the 5-step severity scale developed by Nielsen et al [47], which is a tool widely applied to assess the usability of medical technologies and their impact on patient safety. This severity scale ranges in value from “0” for no usability problem to “4” for usability catastrophe, with mean scores of judgements from multiple evaluators used during heuristic usability evaluation [47].

While heuristic evaluation has the advantage of being more intuitive, efficient, and cheap, with less requirements for advanced planning and involvement of test users [44], it only identifies half of the usability problems that are related to the design of system interfaces [48]. A modified version called heuristic walkthroughs, which also involves the observation of end users, was associated with better detection of usability problems, mainly those characterized as moderate and severe [49]. This has been confirmed by one of the studies that reported heuristic walkthroughs to be effective in the identification of more severe usability problems [43]. Despite the advantages of heuristic evaluation, there are certain limitations associated with this approach. For example, the heterogenous nature of heuristics or guidelines applied in different settings indicates the lack of gold-standard guidelines applicable to every context [50]. Also, because heuristics are broadly defined, they are often interpreted and applied differently by different experts [50]. These limitations highlighted the need to explore alternative approaches of usability evaluation, preferably those that also consider input from end users.

The think-aloud technique is one of the controlled user testing approaches used by multiple studies in our review to evaluate the usability of eDS [33-35,37,43]. This method requires participants to verbalize their impressions about an interface while using it, enabling data collection from both direct observation and users’ self-reported statements [51,52]. This method has the advantage of providing insights into both design and learnability problems associated with systems [50]. Kernebeck et al [35] demonstrated that both effort and performance metrics can be effectively captured using a concurrent think-aloud evaluation approach, and emphasized the critical need to involve actual users from the start of the development process to enable a more transparent evaluation that meets the needs of end users. More importantly, the findings from Watbled et al [43] highlighted that this approach can be successfully integrated with heuristics, offering an advantage of a more holistic assessment of problems from both experts and users.

Eye-tracking is another controlled user testing method used for eDS usability evaluation [40]. In this method, eye-trackers record and analyze information on eye movement, fixation, and screen gaze to assess if the tasks involved are demanding. Questionnaires and semistructured interviews were also used to assess the usability issues with eHRs. Our review identified the use of the SUS [53]—a 10-item Likert tool that provides overall assessment of system usability. The SUS is a nonproprietary self-administered questionnaire with good validity and reliability; however, it is not robust and specific enough in identifying usability issues specific to eHRs.

The usability evaluation techniques used in the included studies, such as the use of heuristics, were not only used to identify issues related to eDS content, such as unnecessary or missing information, poor organization, and inconsistencies in formatting [32,38] but also used to understand the visualizations within eDS systems, including those associated with presenting medication lists and diagnoses [33,35,37,43]. The usability problems identified in the eDS systems had significant consequences, for example, the need for extended deadlines for task completion and errors in patient identification, which ultimately impacted the system's quality and performance [43]. These findings emphasize the importance of improving the speed and quality of systems when designing technologies for use in the context of eDS. It has been proposed that integrating usability testing methods during the development of these systems can potentially reduce adverse health events and outcomes.

In order to provide optimal and safe health services, eDS should provide clinically relevant, accurate, adequate, and clear display of relevant information. The content and quality of discharge summaries have implications for patient outcomes after their discharge from hospital [54]. While technological solutions can significantly improve the content and quality transfer of information, factors such as health literacy and individual patient differences are other important factors to consider during system implementation. This review highlights that this can be achieved through applying rigorous usability assessment techniques that require experts (heuristic evaluation and walkthroughs) approach with a user-based (think-aloud approach) method [50]. However, the limited number of studies assessing the usability of eDS or discharge instructions by patients with different levels of health literacy highlights the need for additional research.

Given the diverse user base of eHRs and discharge summaries in primary care settings, which includes physicians, nurses, pharmacists, and other health professionals, it is crucial to have systems that are easy to navigate, gather and select information, and interpret that information. Therefore, it is important to use a robust usability assessment approach that takes into account the wide range of users, including health professionals and patients, to develop a platform that can be used without significant challenges. Developing systems that can overcome usability issues, such as poor organization and display fragmentation, workflow interference, and cognitive overload, can affect the quality of information required to enable clinical decision making by health professionals and, therefore, continuity of care [55]. With emerging interest around International Patient Summary, which aims to provide a relatively generic means of communication for “unplanned, cross border care” [56], some of the identified usability techniques, especially those applicable to medication and condition summaries, can be used in this broader context.

Although most of the included studies assessed or explored different usability evaluation methods, the usability metrics used were heterogenous in nature. More studies focused on standardized usability metrics like efficiency, effectiveness, and satisfaction, as highlighted in the ISO (International Organization for Standardization) 9241-11 Ergonomics of human-system interaction [17] may shed light into the most effective approach for usability evaluation of eDS. Overall, usability evaluations applied on interfaces should aim to achieve adequate validity, thoroughness, and reliability [57]. In this context, considering the limitations with individual techniques, adopting a multimodal evaluation approach, for example, through combining heuristic evaluation with user testing methods or a questionnaire, may better achieve these objectives. More importantly, there should be an increased focus on developing and implementing usability evaluation techniques that consider factors such as learnability, regular use, error protection, accessibility, and maintainability, as highlighted in the ISO 9241-11 [17]. Another important consideration is the limited geographical locations covered by the included studies, which may limit the applicability of the findings in other settings with different electronic health systems and infrastructures. Lastly, the evidence concerning usability evaluation theories, approaches, and implementation frameworks specific to discharge summaries remains notably scarce. This highlights the need for further research in the area.

Even though we included a range of databases and gray literature sources, it is possible that we may have missed studies indexed in sources not included in this review. Our search strategy was specifically restricted to discharge summaries or instructions, which may have excluded usability evaluation techniques used in the context of EHRs in general. Some of these techniques identified in previous works focusing on EHRs could also be relevant to eDS [28]. We also acknowledge that despite our systematic and thorough approach, the potential for bias exists due to the reliance on a single reviewer for data extraction and quality appraisal.

### Conclusions

We have identified multiple usability evaluation methods that can be used to identify usability concerns applicable to eDS and eDS systems as well as other discharge communication tools. While the evidence in this area is still emerging, especially in terms of standardizing the usability metrics used, published studies indicate the use of a variety of generic methods to effectively assess different aspects of discharge summary contents. These aspects include the presence of necessary information, organization, formatting, as well as the presentation (display and layout) of the systems used to host the eDS.

Heuristic and think-aloud evaluation techniques emerged as the most used methods. They were used either independently or in conjunction with other techniques, such as validated surveys or semistructured interviews. These methods were not only used to identify usability issues with eDS and eDS systems but also revealed severe issues that had implications for the quality and performance of these systems.

## Appendix

Multimedia Appendix 1 Search strategy.

Multimedia Appendix 2 Characteristics of included studies.

Multimedia Appendix 3 PRISMA checklist.

## Abbreviations

eDS: electronic discharge summaries
eHR: electronic health record
ISO: International Organization for Standardization
PRISMA: Preferred Reporting Items for Systematic Reviews and Meta-Analyses
SUS: system usability survey
