# Supplementary material for: Usability Evaluation Methods Used in Electronic Discharge Summaries: Literature Review
Source: J Med Internet Res. 2024 Sep 12;26:e55247. doi: 10.2196/55247 (PMC11427863; doi:10.2196/55247)
Supplement: Multimedia Appendix 1 [file jmir_v26i1e55247_app1.docx]

**APPENDIX 1.** Search strategy

| **PubMed** | | **Search (09/06/23)** |
| --- | --- | --- |
| **#1** | "usability evaluation"[All Fields] OR "usability test"[All Fields] OR "usability testing"[All Fields] OR "usability engineering"[All Fields] OR "usability inspection"[All Fields] | 2171 |
| **#2** | "discharge summar*"[All Fields] OR "discharge communication"[All Fields] OR "continuity of care"[All Fields] OR "transfer of care"[All Fields] OR "clinical handover"[All Fields] OR "electronic discharge"[All Fields] OR "patient discharge"[All Fields] | 51,753 |
| **#3** | #1 AND #2 | 20 |
| **Web of Science** | | |
| **#1** | ((((ALL=(usability evaluation)) OR ALL=(usability testing)) OR ALL=(usability test)) OR ALL=(usability engineering)) OR ALL=(usability inspection) | 47,636 |
| **#2** | ((((((ALL=("discharge summar*")) OR ALL=("discharge communication")) OR ALL=("continuity of care")) OR ALL=("transfer of care")) OR ALL=("clinical handover")) OR ALL=("electronic discharge")) OR ALL=("patient discharge") | 14,176 |
| **#3** | #1 AND #2 | 71 |
| **ACM Digital Library** | | |
| **#1** | [All: "usability evaluation"] OR [All: "usability testing"] OR [All: "usability test"] OR [All: "usability engineering"] OR [All: "usability inspection"] OR [All: ))] | 8612 |
| **#2** | [All: "discharge summar*"] OR [All: "discharge communication"] OR [All: "continuity of care"] OR [All: "transfer of care"] OR [All: "clinical handover"] OR [All: "electronic discharge"] OR [All: "patient discharge"] OR [All: ))] | 319 |
| **#3** | [[All: "usability evaluation"] OR [All: "usability testing"] OR [All: "usability test"] OR [All: "usability engineering"] OR [All: "usability inspection"]] AND [[All: "discharge summar*"] OR [All: "discharge communication"] OR [All: "continuity of care"] OR [All: "transfer of care"] OR [All: "clinical handover"] OR [All: "electronic discharge"] OR [All: "patient discharge"]] | 26 |
| **Medline (via Ovid)** | | |
| **#1** | ("usability evaluation" OR "usability testing" OR "usability test" OR "usability engineering" OR "usability inspection").af. | 2164 |
| **#2** | ("discharge summar*" OR "discharge communication" OR "continuity of care" OR "transfer of care" OR "clinical handover" OR "electronic discharge" OR "patient discharge").af. | 52,186 |
| **#3** | #1 AND #2 | 20 |
| **CINAHL (via EBSCOhost)** | | |
| **#1** | ""usability evaluation" OR "usability testing" OR "usability test" OR "usability engineering" OR "usability inspection"" OR (MH "Usability Study") | 774 |
| **#2** | (MH "Transfer, Discharge") OR (MH "Discharge Planning+") OR (MH "Patient Discharge+") OR (MH "Electronic Health Records+") OR (MH "Continuity of Patient Care+") OR (MH "Clinical Information Systems+") OR ""discharge summar*" OR "discharge communication" OR "continuity of care" OR "transfer of care" OR "clinical handover" OR "electronic discharge" OR "patient discharge"" | 99,268 |
| **#3** | #1 AND #2 | 101 |
| **ProQuest Central** | | |
| **#1** | ("usability evaluation" OR "usability testing" OR "usability test" OR "usability engineering" OR "usability inspection") | 28,234 |
| **#2** | ("discharge summar*" OR "discharge communication" OR "continuity of care" OR "transfer of care" OR "clinical handover" OR "electronic discharge" OR "patient discharge") | 143,656 |
| **#3** | #1 AND #2 | 537 |

**Grey Literature Data Sources and Search Strategy**

The following key terms were used for the grey literature source: “discharge summary”, “electronic discharge summary”, “discharge communication”, “clinical handover”, “transfer of care”, “continuity of care” to locate potentially useful resources. In addition, the websites of international sources which promote safe medication practices, evidence-based recommendations and provide clinical standards were searched. These included the UK National Institute for Health and Care Excellence (NICE), the Institute of Safe Medication Practices (ISMP), the National Quality Forum in the United States (US), The Joint Commission (US) and The Joint Commission International, and the Health Quality and Safety Commission in NZ. Websites of Australian health jurisdictions were also searched for relevant guidelines, procedures, policies of other relevant information.
