# Supplementary material for: Usability Evaluation Methods Used in Electronic Discharge Summaries: Literature Review
Source: J Med Internet Res. 2024 Sep 12;26:e55247. doi: 10.2196/55247 (PMC11427863; doi:10.2196/55247)
Supplement: Multimedia Appendix 2 [file jmir_v26i1e55247_app2.docx]

**APPENDIX 2.** Characteristics of included studies

| **Authors, year, country** | **Study design, setting** | **Participant characteristics and sample size (enrolled)** | **Primary aim(s)** | **Usability evaluation approach(s)** | **Description of discharge summaries/instructions** | **Usability metrics** | **Major findings** | **Conclusions** |
| --- | --- | --- | --- | --- | --- | --- | --- | --- |
| Barton et al 2023 [32]  USA | Qualitative review by experts | Emergency medicine physicians (n=2), nurses (n=3), a geriatrician and an older care partner | To assess a method for integrating diverse expertise such as clinical, patient and care partner, and IT, with human factors engineering expertise in the evaluation of patient-facing emergency department after visit summary (AVS). | Heuristic evaluation (Three staged process) | Two versions of an emergency department AVS | Usability issues identified by human factors engineering experts, clinicians (physicians, nurses) and carer, and an IT expert.  Impact ratings of usability issues on patient comprehension and safety. | 60 unique usability issues with 108 heuristics violations were identified on the following categories: Readability, Minimalism, Comprehensibility, Content and Organisation.  The heuristics violations range from 0 to 16 in number, with clarity of content, absence/lack of information, relevance and group among issues frequently identified.  Among examples of highest rates (largest negative impact) usability issues was the lack of information on whether the medication list was up-to-date or was reviewed by the emergency department. | Conducting a thorough heuristic evaluation with a diverse range of expertise is necessary, especially when patient safety is a concern.  Adopting a staged approach that involves multiple experts for heuristic evaluation can result in a reliable system for identifying usability issues, including those that patients consider significant. |
| Busse et al 2021 [33]  Germany | Qualitative observational study  Paediatric palliative care | Paediatric palliative care (PPC) healthcare professionals (n=20; 11 physicians and 9 nurses) | To evaluate how potential users from the paediatric palliative care setting perceived an electronic cross-facility system. | Think-aloud followed by a semi-structured interview.  Remote evaluation | A web-based system that can be accessed on the internet in real-time of PPC professionals who are given personal access to a patient’s ECH, which was linked to electronic health records used at the palliative paediatric and specialised outpatients. | Perceptions of usefulness  Learnability | Because of the diversity of electronic systems used, automation of transferring data between systems was found critical.  Participants suggested further functions for free text search and content on, for example, information about treatment. They also wished for a better visualisation of the diagnosis sorting, which would make the causality more comprehensible. | Introducing a free text search feature or incorporating 'opening' and 'closing' fields could enhance the usability of the electronic system.  Contents should be formulated in a way that reduces information load and confusion.  Electronic cross-facility system should be clear and facilitate communication among healthcare professionals. |
| Doyle et al 2022 [34]  Australia | Exploratory study  Tertiary paediatric hospital | Parents of children aged 1-12 years with limited knowledge of technology workflows (n=11)  Physicians (n=8) | To understand parent and clinician experience of discharge communication and engagement in clinical research.  To determine the efficacy of 2 different (used for services and research purposes) digital tools integrated into a paediatric patient registry. | Direct observation followed by think-aloud method, followed by a user satisfaction survey (SUS scores) | A web-based digital discharge communication system allowing physicians to choose from disease-specific templates using a desktop interface to create personalized discharge instructions that can be sent to a parent’s mobile phone | Speed, accuracy & understanding of a series of tasks (each task consisted of any combination of one to three actions: start action, find method, and select method)  User satisfaction | Of a total of 121 tasks undertaken by all parents on a mobile interface, 107 (88.4%) were a success, 1 (0.8%) was a failure, no task was ascribed to software failure, and 13 (10.7%) were not observed.  Of 176 tasks observed during interaction with desktop interface, 155 (88%) were a success, 9 (5%) were a failure, 6 (3.4%) were software failure.  The mean satisfaction score (mean SUS score 94/100) for parents on use of mobile interface was high, physicians scored high on the desktop interface (93/100). | The analysis of semi-structured interview responses was useful in identifying communication challenges related to discharge communication.  Usability testing methods focusing on software interface and the utilisation of the SUS helped uncover potential issues that can be addressed for users of digital tools. |
| Kernbeck et al 2022 [35]  Germany | Qualitative observational study  Paediatric palliative care | Paediatric palliative care professionals work in children’s and adolescents’ hospital (n=14; 10 nurses and 4 physicians) | To evaluate the acceptance of the medication module from the perspective of potential users in paediatric palliative care and to involve them in the development process. | Think-aloud (screen movements and audio were recorded during observation for analysis) followed by semi-structured interview | A medication module containing two key elements: (1) a view for the prescription of medications and (2) a view for the confirmation of medication administration. Both modules were integrated into a software and were used for adult palliative care. | Performance & effort expectancy  Learnability | Two main categories identified: performance expectancy (e.g., clarity and readability of medication documentation) and effort expectancy (e.g., precise information of medication dose)  Precision around medication information including doses can improve usability.  Display fragmentation (when some information is found outside of display window) may have a negative effect on cognitive load of users.  Lesser data entry requirements may reduce confusion and usability concerns. | The study identified key factors that influence user acceptance in the context of performance and effort expectancy.  Emphasis should be given on the reduction of display fragmentation to reduce cognitive load on users. |
| Naik et al 2017 [36]  USA | Observational (A multiphasic user-centred design)  Medical centre | Patient end-users (people with colorectal cancer) (n=7; aged 49-73 years)  Human factors experts (n=1) | To transform physician-centred discharge warnings into an understandable, usable format for patients using health literacy and usability heuristics standards and cognitive interviews | Heuristic evaluation (Nielsen’s) | A discharge instruction containing warning symptoms relevant to people with colorectal cancer.  Health literacy expert reviewed the readability of the discharge instruction to achieve a reading level to that of an 8^th^ grader. | Unclear | A comprehensive heuristic evaluation revealed several inconsistencies regarding the presentation and readability of the information provided by the discharge warnings.  The human factors expert integrated the consensus findings of the heuristic evaluation into a revised version of the discharge warnings lexicon page using a simpler language base and a more consistent design | Patient-centred design of discharge instructions are easily understood.  The structure and content of discharge warnings are critical in activating patients to monitor their health and communicate with clinicians.  It was unclear from findings if the instructions were administered separately or as part of the overall discharge summary. |
| Soto et al 2019 [37]  Canada | Mixed-methods study  Community setting | Medical professionals (n=12; 9 general practitioners and 3 family medicine residents) | To improve health information exchange and use of clinical information for decision making | Heuristic evaluation of end-users followed by think-aloud observational sessions | Three eHR systems widely used in Quebec province were chosen. These systems have a vendor-mediated health information exchange (HIE) integration feature.  The eHR-HIE interface containing information on clinical data domains (medications, pathology findings, and images from medical imaging examinations) | Performance  Perceptions of usefulness, benefits, and barriers | The main usability issues included significance of codes, workload, and guidance problems, with most of the problems deemed to have minor severity level.  Drug prescription was the domain with the highest number of usability problems (43%), and visualisation of the medication list was the most problematic feature.  All physicians perceived that the eHR-HIE integration met the intended goal for which it was designed, including facilitating access to clinical data. | Clinicians identified where heath information exchange system is most useful in their practice, which was mainly around medications.  Addressing usability problems can improve integration of electronic health records and health information exchange. |
| Tremoulet et al 2018 [38]  USA | Qualitative evaluation of medical documents  Two hospitals with two different eHRs | Four teams each containing a human factors expert experienced in heuristic evaluations and a clinical expert without heuristic evaluation experience | To conduct heuristic evaluation to identify potential usability problems and their level of severity | Heuristic evaluation (Nielsen’s) | Simulated after-visit summaries populated with patient data generated from EHRs of two hospitals | Content, comprehensibility, structure, and readability issues  Severity of usability problems identified | Content issues were the most prevalent usability problems, while problems were also identified around readability (e.g., poor contrast, unclear layout and changes in font), comprehensibility, and organisational issues.  Multiple content, comprehensibility, readability, and organisational issues were deemed catastrophic in severity (e.g., lack of clear medication list, confusing order of information, formatting and issues of page breaks contributing to problems of grouping information). | The study identified a range of usability issues affecting coordination of care, such as unnecessary/missing information, poor organisation, and inconsistent formatting.  Heuristic evaluation is a quick and inexpensive approach to evaluate electronic records including any transfer of information. |
| Tremoulet et al 2021 [39]  USA | A literature review with mixed methods  Two hospitals with two different eHRs used to generate simulated discharge summaries | Outpatient care providers comprising primary care physicians, nurse practitioners, directors of nursing, social workers, transition-of-care nurses, and medical directors (n=15 for interview and n=58 for survey) | To provide insight into how existing acute care eDS in the United States support outpatient providers in coordination of care of older adults | Heuristic evaluation (Nielsen’s) with interview and survey | Simulated eDS from two hospitals which use different eHR vendors, were developed using de-identified data of older patients | Unclear | Several usability issues were identified and rated as moderate or above severity (e.g., no indication on whether medications were temporary or permanent, medication frequency missing etc.)  A range of recommendations that are software- and human-centred were proposed by the researchers. | The current heterogeneity among discharge summaries and the different eHR vendors makes it difficult for outpatient providers to coordinate care for recently discharged patients.  Standardised discharge summaries across acute care settings could improve care coordination. |
| Vaigneur 2015 [40]  USA | Experimental and survey  Community setting | Novice (people with no prior experience with the discharge summary) readers (caregivers) of discharge instructions (n=74; aged 18-25 years) | To examine how adjusting readability level of discharge instructions (through formatting changes) affect a user’s ability to understand and recall information from the discharge instructions | Eye-tracking (experimentation using an eye-tracker) followed by System Usability Survey (SUS) | A modified discharge instruction, where original format was modified for better readability (to the level of 8^th^ grade or below).  The original discharge instructions contained the mandatory items per the US Joint Commission on Accreditation of Healthcare Organisation, which are reason for hospitalisation, significant findings, procedures, and treatments provided, discharge conditions, patient and family instructions, attending physician’s signature). | Accuracy & efficiency (of comprehension and recall of discharge instructions) | Based on the eye-tracking experiment, the high readability discharge instructions received more visual attention than the original format discharge instructions.  The modified discharge instruction was also associated with lesser duration of screen gazes and better comprehension of instructions.  Participants required less mental demand when using the high readability discharge instructions compared to low readability discharge instructions. | Through improving patients and their caregivers’ comprehension of discharge by improving the readability and layout of the designs, discharge instructions can lead to improved health management.  The findings indicate the importance of considering readability level and layout in discharge instructions. |
| Walsh et al 2016 [41] – I  Australia | Qualitative evaluation of My Health Record (MyHR) | Domain (MyHR) and heuristic experts/evaluators (n=3) | To identify potential usability issues within MyHR focusing on e-health literacy | Heuristic evaluation (Nielsen’s) | Australia’s electronic personal health record system (MyHR) | Unclear | The heuristic evaluation revealed various usability violations on both the information website and the personal health record components.  The evaluation revealed a range of potential usability problems that may affect MyHR users, including lack of use of plain language and translated resources, website navigation, design elements and registration processes. | Several elements of the MyHR have usability problems that particularly affect people with low e-health literacy.  Usability concerns with language use, display and layout, lack of audio-visuals and translated services are among barriers with potential effect on people with low health literacy. |
| Walsh et al 2021 [42] – II  Australia | Qualitative evaluation of MyHR | Domain (MyHR) and heuristic experts/evaluators (n=3) | To identify usability issues with MyHR through an updated heuristic evaluation. | Heuristic evaluation (Nielsen’s) | Australia’s electronic personal health record system (MyHR) | Unclear | Of the 37 heuristics criteria, there are multiple violations that are rated as simple to resolve (e.g., adding an introductory section on the home page of the record) to violations that may be challenging to improving (e.g., deprioritising health professional-targeted search results made on the MyHR website).  Despite the improvements in the information quality presented in MyHR, there remain unmet needs for people with or at risk of poor ehealth literacy around information and usability. | Although there are some improvements on MyHR in terms of presentation and information quality in consideration of culturally and linguistically diverse audiences, there remains some usability concerns in people with low health literacy levels, which may affect access and use of MyHR. |
| Watbled et al 2018 [43]  France | Observational and qualitative design  Hospital | Human factors experts (n=2) for heuristic evaluation  Medical professionals (14 physicians & 8 medical secretaries) for observational usability testing | To apply a combination of methods for longitudinal usability evaluation throughout the system development lifecycle.  To identify causes of usability flaws. | Heuristic evaluation (Sears’) followed by a laboratory-based user testing and *in situ* observations of technology use | Two systems were assessed.  *System #1*: was a voice recorder plugged into a computer that was connected directly to the patient’s eHRs.  *System #2*: was a wireless voice recorder that enabled a summary to be dictated anywhere. | Consequences of usability flaws | The usability evaluation methods identified multiple system flaws, with the usability flaws identified using the heuristic evaluation confirmed by the laboratory user testing approach.  Field observations identified the same usability flaws as those identified using the heuristic evaluation and user testing. | The use of a combination of usability evaluation methods enabled identification of several major usability flaws.  The findings highlight the importance of conducting a thorough analysis of the context of use – for instance, the entire discharge summary production process. |

eDS, electronic discharge summaries; EHRs, electronic health records; MyHR, My Health Record; PPC, paediatric palliative care
